# Strengthening dermatology capacity and competency in resource-limited settings: A viewpoint

**DOI:** 10.1371/journal.pntd.0013720

**Published:** 2025-11-21

**Authors:** Annika B. Wilder-Smith, Jianing Li, L. Claire Fuller, Sarah N. Anwar, Emma van Rooijen, Esther E. Freeman, Michele E. Murdoch

**Affiliations:** 1 Faculty of Medicine, University of Zurich, Zurich, Switzerland; 2 International League of Dermatological Societies, London, United Kingdom; 3 Department of Dermatology, Massachusetts General Hospital, Boston, Massachusetts, United States of America; 4 West Herts Teaching Hospitals NHS Trust, Watford, United Kingdom; Albert Einstein College of Medicine, UNITED STATES OF AMERICA

## Abstract

Skin diseases are among the most common health conditions in low-resource-limited settings (RLS), yet dermatology remains an underserved specialty. The Global Burden of Disease Study 2021 ranked skin diseases among the top ten causes of non-fatal disease burden worldwide, but many RLS still face a severe shortage of dermatologists and dermatology training, alongside underdeveloped health infrastructure, and restricted access to diagnostics, treatments, and medications. In recognition of the impact of skin conditions on overall health and wellbeing, the World Health Assembly (WHA) passed a resolution prioritizing skin health as a global health priority earlier this year. The resolution aims to advance universal health coverage and strengthen primary care systems, recognizing that access to basic skin care is essential for achieving health equity. The WHO Neglected Tropical Diseases (NTD) Working Group on Capacity Strengthening and Training has developed a framework to guide the dissemination of dermatology resources. This framework emphasizes identifying the correct target audiences, including healthcare professionals, health ministry staff, community members, and people affected by skin conditions, with tailored tools and techniques for each group. Tools include hybrid models (both online and in-person) that are contextually appropriate, while techniques focus on sustainability, certification incentives, and ensuring resources are free, accessible, and maintain trust and quality through a grassroots approach. This article highlights key dermatological resources available to healthcare workers in RLS, with a focus on the *Community Skin Health Journal*. The journal’s reach, usage, and effectiveness are evaluated, along with its recent challenges. Finally, we propose strategies to improve access and dissemination, ensuring that dermatology education and capacity strengthening efforts in RLS are impactful, scalable, and sustainable, with the goal of improving skin health for all.

## Introduction

Providing dermatological care in resource-limited settings (RLS) presents significant challenges for frontline healthcare workers. Although skin diseases are among the most common health conditions in RLS and the Global Burden of Disease Study 2021 ranked them among the top 10 causes of non-fatal disease burden worldwide [1], dermatology remains an underserved specialty. RLS often lack dermatologists, have underdeveloped infrastructure, and face resource constraints such as limited access to medications, diagnostics such as dermatopathology and treatment options [2]. Since few high-quality publications exist in RLS, there is little data on the true impact of dermatological conditions in these regions.

There is a great need to strengthen both capacity (workforce numbers) and competency (knowledge or training) of healthcare staff for skin conditions in RLS. Not only are there few dermatologists in RLS, but they mainly work in urban areas and rarely travel to rural communities. Sub-Saharan Africa has only 0–3 dermatologists per million population [2]. Those living in rural areas struggle to access specialist advice due to transport and other out-of-pocket expenses [3]. This gap in specialized care provision exacerbates the burden on primary healthcare workers. They are often the first and sometimes only point of contact for patients with skin-related issues, but frequently lack sufficient training in dermatology.

The World Health Organization (WHO) Neglected Tropical Diseases (NTD) 2021–2030 Roadmap highlighted the extensive unmet burden of *all* skin diseases in communities [4]. The recently adopted 78th World Health Assembly (WHA) resolution on “Skin diseases as a global public health priority” emphasized the importance of accelerating programmatic action to reduce disability and death from skin diseases in order to help achieve Universal Health Coverage and urged Member States “to strengthen competency-based education for the health workforce in primary care settings in the identification and management of skin diseases and their co-morbidities” [5]. The strategy towards achieving the resolution encouraged “capacity-building and training for healthcare professionals and workers on skin diseases, including through the WHO Academy and other technical training platforms, and identification of centers of excellence, including WHO collaborating centers, in various WHO regions” [5]. Capacity- and competency-building can be executed through the distribution of teaching and training materials. Available materials will be discussed, with an emphasis on the Community Skin Health Journal, one example of a journal that can help to facilitate the WHA resolution.

## Available teaching materials and resources

Materials to support healthcare workers in dermatology range from printed to digital resources. International organizations, such as the International League of Dermatological Societies (ILDS) have developed training materials and educational webinars, to address gaps in dermatological knowledge [6]. Such materials, however, may not reach the areas of need, where internet connectivity can be unreliable and literacy levels can vary [7].

The WHO has also spearheaded initiatives to train primary healthcare providers to identify and manage skin conditions. Among these was the OpenWHO online learning platform, which included a dedicated channel for courses pertaining to NTDs [8]. Courses were created on every type of skin NTD, and many were translated into additional languages. As of December 2024, nearly 82,000 learners had accessed skin-related online courses via OpenWHO, with the most highly frequented course on tropical dermatology (*n* = 11,838). The skin NTD courses have transitioned to a new teaching platform called the WHO Academy [9]. InfoNTD is another notable platform which provides free access to hundreds of tools, online courses, and publications focused on cross-cutting topics related to NTDs [10]. The global demand for such resources is evident as Infolep, a website dedicated to resources exclusively on leprosy, is used by more than 22,000 people across 185 countries [11]. However, many freely available online resources are only provided in English, thus limiting their reach.

The future of dermatological education in RLS depends on improving the quality and distribution of resources for both skin NTDs and other general skin diseases. This involves analyzing existing training materials to assess content and quality and identifying critical gaps to set priorities for future resource development. It will also be important to develop appropriate dissemination strategies.

Hybrid models, which include remote or self-directed learning, together with in-person training with on-going mentorship are an ideal way to improve capacity-building and staff competency in RLS [12]. Although there is a wealth of dermatological training materials available, they need to be easily accessible and relevant to RLS. The WHO Skin NTDs Capacity Strengthening and Training Working Group developed a framework to guide such initiatives and identified three key elements: The correct target audience, correct context, and correct techniques.

Firstly, clarifying the target audience is key, as training information for healthcare professionals will be different from that required for community leaders, health ministries, or affected persons. Each group requires unique engagement strategies based on their access to resources, background literacy, and impact on public health practices and policies. Secondly, understanding the local context for appropriate dissemination is needed [13]. Thirdly, successful interventions depend upon correct techniques, such as culturally sensitive community outreach programmes, engaging methods, and professional incentives for health workers. A grassroots approach that is tailed to local contexts and cultures can help rebuild trust and encourage participation in educational and engagement activities, such as collaborations with local leaders, traditional healers, teachers, youth groups, and women’s groups. Furthermore, involving people affected by NTDs fosters advocacy, reduces stigma, and raises awareness. Healthcare professionals benefit from ongoing professional development and may be incentivised through certification and accreditation of training.

## Case study: *Community Skin Health Journal*

One initiative for strengthening dermatological competency is the *Community Skin Health Journal (CSHJ),* an exemplary teaching journal designed to meet the needs of frontline healthcare workers in RLS [14]. The journal’s production and distribution are funded by the ILDS/International Foundation for Dermatology (IFD), and its voluntary international team includes qualified dermatologists serving as Editorial Board members, translation-checkers, and the Editor. Founded in 2004 by Dr. Paul Buxton, and previously known as the *Community Dermatology Journal*, it was renamed in 2019 to reflect its broader focus on promoting global community skin health through education. The journal aims to bridge the knowledge gap for healthcare workers in RLS and also serves as a valuable resource for trainee dermatologists with an interest in global health and tropical dermatology. It encourages submissions from low- and middle-income settings to promote the development of contextually relevant and practical resources for healthcare professionals worldwide.

*CSHJ* is a freely available, bi-annual publication in six languages—English, Spanish, Portuguese, French, Simplified Chinese, and Arabic. It can be freely downloaded via the ILDS website (https://www.ilds.org/what-we-do/project-and-programme/community-skin-health-journal/) or the *CSHJ* app (available at https://apps.apple.com/gb/app/community-skin-health-journal/id1398917203 and https://play.google.com/store/apps/details?id=org.ilds.cdj.app&hl=en_NZ) or posted as hard copy. It is indexed on GoogleScholar, the World Health Organization Knowledge Action Portal (KAP) on Noncommunicable Diseases (https://knowledge-action-portal.com/en/about), and on the infoNTD platform. Articles are written in non-academic format, with a variety of topics relevant to healthcare providers in RLS (Table 1). Article types include educational pieces, journal club, and quizzes.

**Table 1:** Range of topics covered by *CSHJ*, 2004–2024.

Topics	Frequency
Community Dermatology	34
Skin NTDs	26
Other	22
HIV-Related	12
Wound Management	7
Practical Therapeutics	7
Fungal Infections	6
Dermatology Networks	5
Albinism	4
Insect and Snake Bites	8
Sexually Transmitted Infections	4
Vitiligo	4
Skin Ulcers	3
Drug Eruption	3
Lymphoedema	3

Up until 2024 the total journal print run per issue reached 10,100 English and 750 French copies, reaching over 184 countries. From 2006 onward, an innovative distribution model was also developed, courtesy of the WHO/African Programme for Onchocerciasis Control (APOC). Under this system, journals were posted in bulk to National Onchocerciasis Coordinators within Ministries of Health of selected African countries for onward distribution to primary health centers (e.g., with supplies of ivermectin). When APOC closed in 2015, some coordinators continued this practice, while others transitioned to similar arrangements with their respective National NTD Programme Managers.

**Fig 1:**
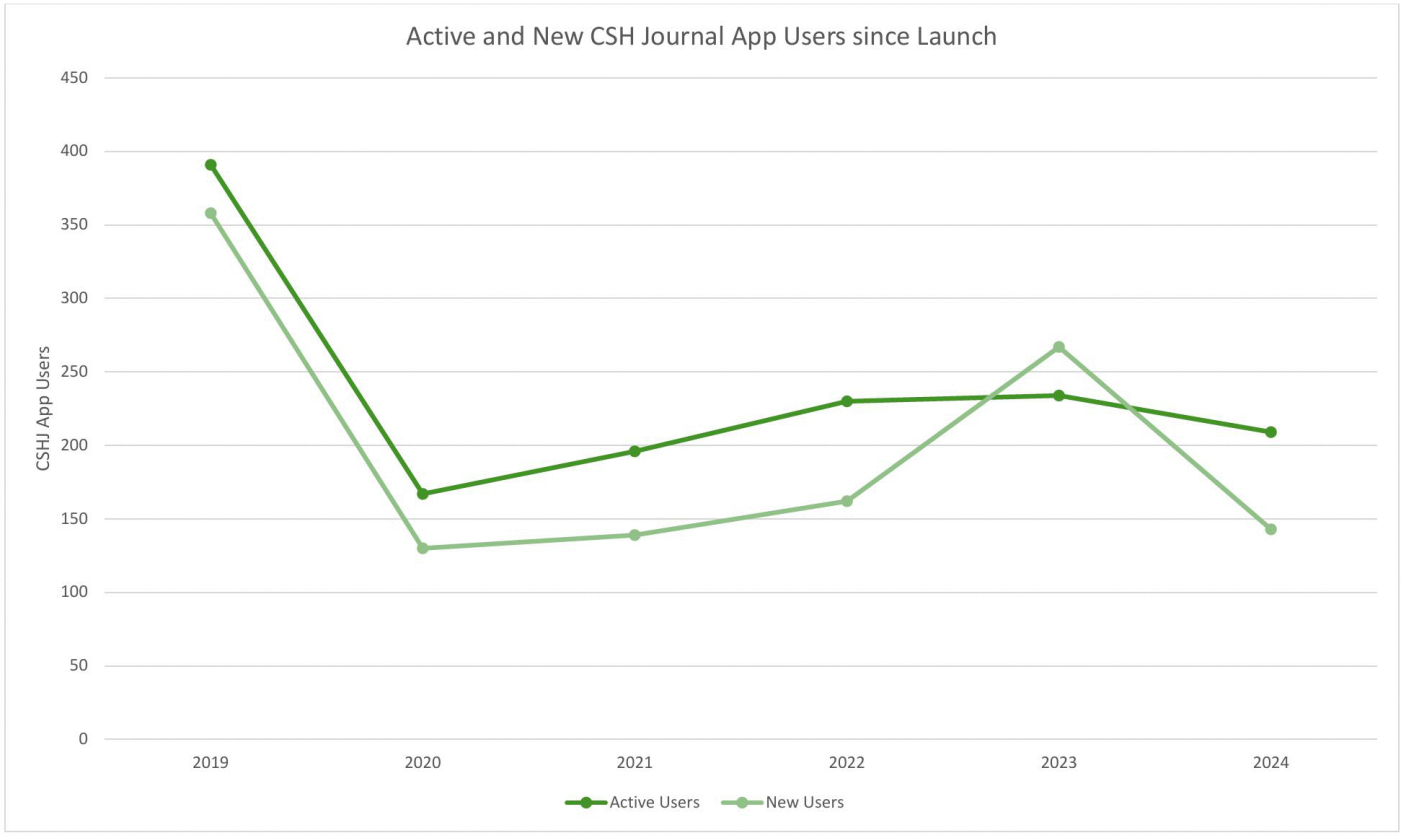
Active and new *Community Skin Health Journal (CSHJ)* app users since launch on 08 December 2018.

Over the years, *CSHJ* has expanded to include electronic formats. As demonstrated in Fig 1, since the app’s launch in December 2018, there have been 1,143 unique active users from 119 different countries, with an average engagement time of almost 52 minutes in 2024. In 2024, there were 143 new users, and 487 new journal views. Regarding the *CSHJ* web pages, which are embedded within the ILDS/IFD website, there was a 97% increase in users from 2023 (*n* = 679) to 2024 (*n* = 1336). Contributors are encouraged to submit their articles to CSH@ilds.org.

## Challenges for *CSHJ*

Rising postal costs have made it expensive to maintain large-scale dissemination of free hard copies through collaborating National NTD Programme Managers, shifting the focus to electronic versions. As a result, RLS with limited internet access will remain neglected. There has also been low engagement to date with the non-English translated versions, possibly because hard copies were only ever printed in English and French, limited awareness of the journal in non-English-speaking regions, and a lack of online access. To tackle this, the freely available *CSHJ* app offers downloadable issues, so, once downloaded in an area with internet, they are accessible if internet connectivity is later unavailable. ILDS Board members are also being actively encouraged to promote the translated journals within their respective countries. Furthermore, translations into other local languages, such as Swahili, are also under consideration.

## Conclusions

The WHO Skin NTD Working Group’s Capacity Strengthening and Competency Framework aims to build dermatology capacity in RLS by identifying the right audiences and delivering context-appropriate tools and techniques. *CSHJ* exemplifies the potential of targeted educational resources to improve dermatology competency of primary healthcare staff in RLS. Its innovative and wide-ranging distribution model, with free print, online, and mobile access, has enabled it to reach healthcare workers globally. Raising awareness about *CSHJ* and other training resources, along with fostering collaboration among healthcare providers, non-governmental organizations, and Ministries of Health is crucial in order to deliver the World Health Assembly resolution and strengthen dermatology competency in RLS.
